# Supplementary material for: Exclusive breastfeeding can attenuate body-mass-index increase among genetically susceptible children: A longitudinal study from the ALSPAC cohort
Source: PLoS Genet. 2020 Jun 11;16(6):e1008790. doi: 10.1371/journal.pgen.1008790 (PMC7289340; doi:10.1371/journal.pgen.1008790)
Supplement: S1 Text — (DOCX) [file pgen.1008790.s001.docx]

**Supplementary Methods**

## Derivation of the mixed effects models

Let $y_{ij}$ denotes the longitudinal BMI measurements for the *i*th individual at *j*th age $t_{ij}$, a child’s BMI can be expressed in the linear mixed-effects model framework as follows:

$y_{ij}=\left\{ sp\left( t_{ij} \right)*{EXBF}_{i}*{GRS}_{i} \right\}{\times\beta}_{sp\left( t_{ij} \right)*{EXBF}_{i}*{GRS}_{i}}+X_{ij}\times\beta_{X}+Z_{i}{\times b}_{i}+\varepsilon_{ij}$, (1)

where $sp\left( t_{ij} \right)$ denotes the cubic spline function of age with two knots (A.3) that is used to catch the nonlinear BMI growth curves, $X_{ij}$ represents the control variables including gestational age, mother’s BMI, education and smoking status during pregnancy, and family income. The term “$sp\left( t_{ij} \right)*{EXBF}_{i}*{GRS}_{i}$” refers to the three-way interactions of spline function of age, EBF and GRS. The $\beta$*'s* are fixed effects and $b_{i}$ is a vector of random effects including random intercept and random slopes of age and age-quadratic term.

## Spline functions

The cubic splines bases were used to catch the peaks and valleys of the children's BMI growth trajectories without sharp corners. Let $sp\left( t \right)$ denote the spline function of age *t* and $\beta_{t}$ denotes a vector of regression coefficients of $sp(t)$, the cubic spline basis with *k* knots $\mathcal{K}_{i}, i=1,\ldots,k$ can be written as:

$${sp(t)\beta}_{t}=\beta_{1}t+\beta_{2}t^{2}+\beta_{3}t^{3}+\sum_{i=1}^{k} \beta_{3+k}{(t-\mathcal{K}_{i})}_{+}^{3}$$

where

$${(t-\mathcal{K}_{i})}_{+}=\left\{ \begin{aligned} t-\mathcal{K}_{i} if t> \mathcal{K}_{i} \\ 0 if t\leq\mathcal{K}_{i} \end{aligned} i=1,\ldots,k. \right.$$

Below figure represents the S-shaped BMI growth trajectory with a peak around age 1 and a valley at age 5-6 for a typical child. Thus, three knots at age 1, 5 and 10 were initially selected to model the children's different growth stages at infancy (0-1 year), toddlerhood and preschoolers (1-5 years), elementary school age (5-11 years) and puberty (11+ years). The likelihood ratio test and Akaike Information Criterion were used to select the optimal knots and the number of knots. The optimal knots were ($\mathcal{K}_{1}$=0.7, $\mathcal{K}_{2}$=1.5, $\mathcal{K}_{3}$=10) for boys and ($\mathcal{K}_{1}$=0.9, $\mathcal{K}_{2}$=1.5, $\mathcal{K}_{3}$=10) for girls.


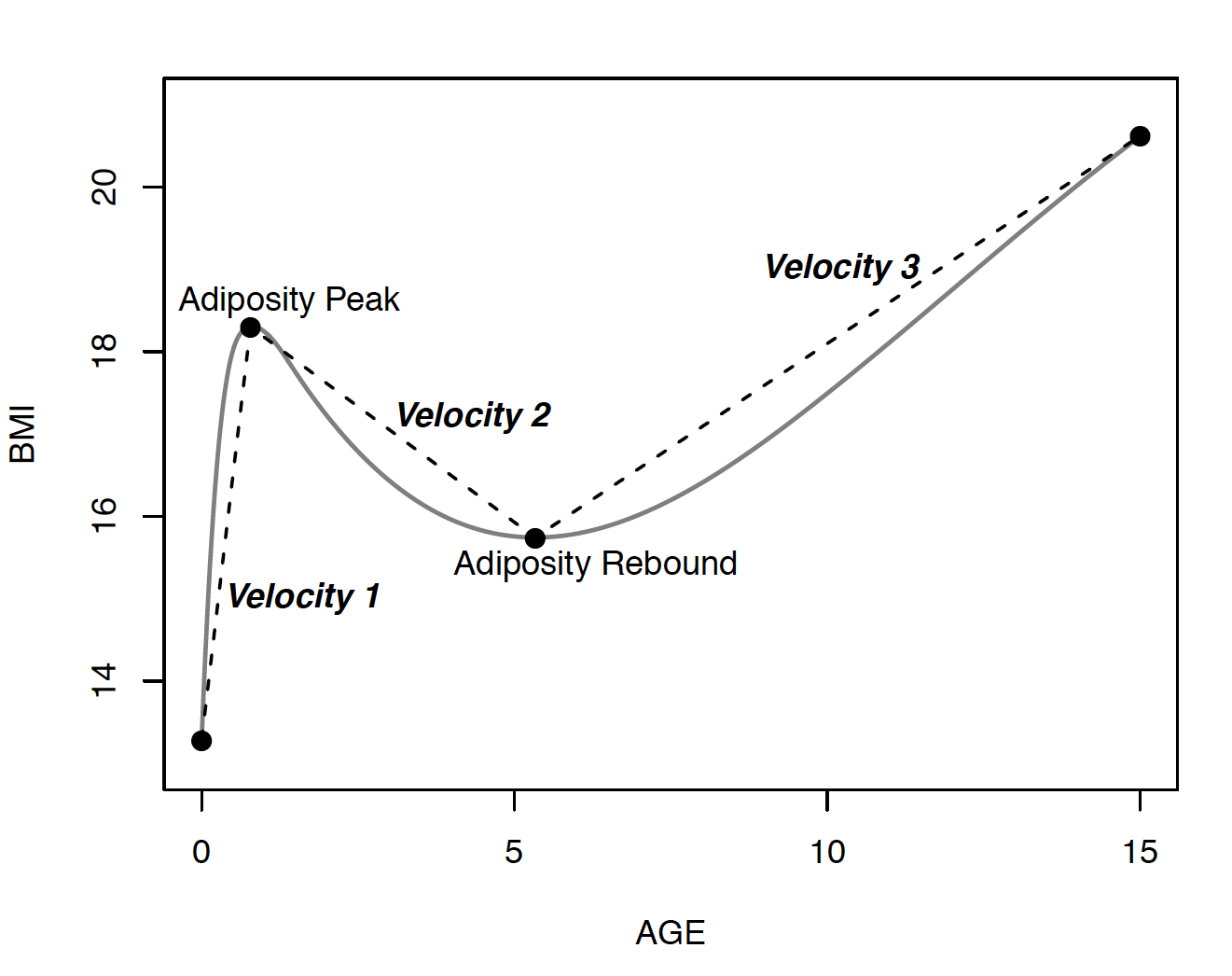


## Variable section and generalized hypothesis testing

The age-specific difference for various GRS and EBF values are estimated using the General Linear Hypothesis (GLH) approach.[1] The GLH test is based on the normal approximation for maximum likelihood estimators using the estimated variance-covariance matrix. The hypothesis can be specified through a constant matrix *L* to be matched with the fixed effects of the model such that $H_{0}:L\beta=m$ where the *m* is the hypothesized value. The estimate of fixed effects $\beta$ follows asymptotically a multivariate normal distribution $\hat{\beta}\sim N(\beta, cov\left( \hat{\beta} \right))$ based on Central Limit Theorem such that the linear form $L\hat{\beta}$also follows asymptotically a multivariate normal distribution: $L\hat{\beta}\sim N\left( L\beta, Lcov\left( \hat{\beta} \right)L^{'} \right).$ Therefor the *p*-value and the 95% confidence interval for the hypothesized value can be obtained accordingly.

For example, to estimate and test the 5 months of EBF effect at age 10 for boys, the *L* matrix is$L\hat{\beta}=L_{2}^{'}\hat{\beta}-L_{1}^{'}\hat{\beta}$ where $L_{2}^{'}\hat{\beta}$ is the estimated mean BMI taking value of age = 10 and EBF = 5, while $L_{1}^{'}\hat{\beta}$ taking EBF = 0, holding all other covariates constant.

1. McDonald L. Tests for the General Linear Hypothesis Under the Multiple Design Multivariate Linear Model. The Annals of Statistics. 1975;3(2):461-6.
