# Supplementary material for: Exclusive breastfeeding can attenuate body-mass-index increase among genetically susceptible children: A longitudinal study from the ALSPAC cohort
Source: PLoS Genet. 2020 Jun 11;16(6):e1008790. doi: 10.1371/journal.pgen.1008790 (PMC7289340; doi:10.1371/journal.pgen.1008790)
Supplement: S1 Table — GRS scores were created for boys and girls separately by scaling the sum of the weighted SNP effects. The weights were beta coefficients obtained from stratified meta-analysis of genome-wide association studies of BMI for men and women in ∼700000 individuals of European ancestry (GIANT consortium and the UK Biobank). (DOCX) [file pgen.1008790.s002.docx]

|  |  |  | Tested Allele | Other Allele | Men | | | Women | |  |
| --- | --- | --- | --- | --- | --- | --- | --- | --- | --- | --- |
| CHR | POS | SNP |  |  | BETA | *p*-value | BETA | | *p*-value | |
| 1 | 49589847 | rs657452 | A | G | 0.0177 | 1.6E-13 | 0.0203 | | 1.1E-19 | |
| 1 | 50559820 | rs11583200 | T | C | -0.0158 | 3.6E-11 | -0.0147 | | 3.4E-11 | |
| 1 | 72751185 | rs3101336 | T | C | -0.0315 | 2.5E-39 | -0.0226 | | 2.0E-24 | |
| 1 | 72765116 | rs2568958 | A | G | 0.0311 | 1.4E-38 | 0.0227 | | 1.3E-24 | |
| 1 | 74991644 | rs1514175 | A | G | 0.0164 | 3.3E-12 | 0.0202 | | 3.9E-20 | |
| 1 | 75002193 | rs12566985 | A | G | -0.0181 | 1.1E-14 | -0.0208 | | 1.4E-21 | |
| 1 | 78446761 | rs12401738 | A | G | 0.0175 | 5.5E-13 | 0.0175 | | 7.4E-15 | |
| 1 | 96924097 | rs11165643 | T | C | 0.0201 | 1.7E-17 | 0.0181 | | 1.7E-16 | |
| 1 | 96944797 | rs1555543 | A | C | -0.0204 | 5.7E-16 | -0.018 | | 6.3E-15 | |
| 1 | 110154688 | rs17024393 | T | C | -0.0711 | 1.7E-23 | -0.0612 | | 3.6E-20 | |
| 1 | 177889480 | rs543874 | A | G | -0.036 | 3.7E-35 | -0.0576 | | 8.7E-100 | |
| 1 | 201784287 | rs2820292 | A | C | -0.0186 | 1.5E-15 | -0.0179 | | 2.3E-16 | |
| 2 | 622827 | rs2867125 | T | C | -0.0577 | 2.4E-77 | -0.0575 | | 1.6E-88 | |
| 2 | 632348 | rs13021737 | A | G | -0.0588 | 8.4E-80 | -0.0577 | | 1.0E-88 | |
| 2 | 25150296 | rs10182181 | A | G | -0.0273 | 5.5E-32 | -0.0362 | | 5.0E-63 | |
| 2 | 25158008 | rs713586 | T | C | -0.0275 | 1.4E-28 | -0.0354 | | 7.0E-55 | |
| 2 | 26928811 | rs11126666 | A | G | 0.0149 | 1.9E-08 | 0.0041 | | 9.5E-02 | |
| 2 | 59302821 | rs887913 | A | G | 0.0088 | 2.0E-03 | 0.0078 | | 2.8E-03 | |
| 2 | 59305625 | rs1016287 | T | C | 0.0225 | 1.3E-18 | 0.0215 | | 4.4E-19 | |
| 2 | 63053048 | rs11688816 | A | G | -0.0096 | 4.5E-05 | -0.0148 | | 9.7E-12 | |
| 2 | 142959931 | rs2890652 | T | C | -0.0215 | 1.8E-10 | -0.015 | | 1.2E-06 | |
| 2 | 143043285 | rs2121279 | T | C | 0.019 | 4.7E-08 | 0.0122 | | 1.9E-04 | |
| 2 | 181550962 | rs1528435 | T | C | 0.0154 | 1.7E-10 | 0.0162 | | 7.2E-13 | |
| 2 | 213413231 | rs7599312 | A | G | -0.0218 | 1.5E-16 | -0.0159 | | 1.1E-10 | |
| 3 | 25106437 | rs6804842 | A | G | -0.0182 | 1.3E-14 | -0.0106 | | 1.7E-06 | |
| 3 | 61236462 | rs2365389 | T | C | -0.0177 | 5.3E-14 | -0.0165 | | 6.0E-14 | |
| 3 | 81792112 | rs3849570 | A | C | 0.0149 | 2.6E-09 | 0.0111 | | 1.6E-06 | |
| 3 | 85807590 | rs13078960 | T | G | -0.024 | 3.0E-16 | -0.0231 | | 5.4E-17 | |
| 3 | 85884150 | rs13078807 | A | G | -0.0235 | 2.2E-15 | -0.0222 | | 8.1E-16 | |
| 3 | 141275436 | rs16851483 | T | G | 0.0355 | 1.6E-12 | 0.0363 | | 2.3E-15 | |
| 3 | 185824004 | rs1516725 | T | C | -0.0383 | 1.4E-28 | -0.034 | | 2.1E-26 | |
| 3 | 185834290 | rs7647305 | T | C | -0.0308 | 7.5E-27 | -0.0271 | | 3.4E-24 | |
| 4 | 45182527 | rs10938397 | A | G | -0.0349 | 2.6E-49 | -0.0315 | | 1.7E-45 | |
| 4 | 103188709 | rs13107325 | T | C | 0.0495 | 3.2E-26 | 0.0452 | | 9.3E-25 | |
| 4 | 145659064 | rs11727676 | T | C | 0.0204 | 1.6E-06 | 0.0153 | | 1.2E-04 | |
| 5 | 75015242 | rs2112347 | T | G | 0.0239 | 8.1E-23 | 0.0309 | | 2.1E-42 | |
| 5 | 124332103 | rs4836133 | A | C | 0.0139 | 6.6E-08 | 0.0134 | | 1.9E-08 | |
| 6 | 34563164 | rs205262 | A | G | -0.0223 | 1.9E-17 | -0.0304 | | 2.1E-35 | |
| 6 | 50803050 | rs987237 | A | G | -0.0453 | 1.3E-49 | -0.0388 | | 1.7E-42 | |
| 6 | 50845490 | rs2207139 | A | G | -0.0442 | 5.1E-46 | -0.04 | | 2.4E-43 | |
| 6 | 108977663 | rs9400239 | T | C | -0.0134 | 7.9E-08 | -0.0188 | | 1.5E-15 | |
| 6 | 163033350 | rs13191362 | A | G | 0.0265 | 2.3E-13 | 0.0208 | | 6.4E-10 | |
| 7 | 75163169 | rs1167827 | A | G | -0.0217 | 2.9E-19 | -0.0197 | | 8.2E-19 | |
| 7 | 76608143 | rs2245368 | T | C | -0.0328 | 4.4E-23 | -0.0173 | | 3.1E-08 | |
| 8 | 76806584 | rs17405819 | T | C | 0.02 | 5.1E-15 | 0.0218 | | 7.8E-20 | |
| 8 | 85079709 | rs2033732 | T | C | -0.0139 | 2.9E-07 | -0.012 | | 1.6E-06 | |
| 9 | 15634326 | rs4740619 | T | C | 0.0187 | 1.5E-15 | 0.0196 | | 3.0E-19 | |
| 9 | 28414339 | rs10968576 | A | G | -0.0233 | 1.1E-20 | -0.0251 | | 1.7E-26 | |
| 9 | 111932342 | rs6477694 | T | C | -0.014 | 8.9E-09 | -0.0126 | | 3.4E-08 | |
| 9 | 120378483 | rs1928295 | T | C | 0.0118 | 5.2E-07 | 0.016 | | 2.8E-13 | |
| 9 | 129460914 | rs10733682 | A | G | 0.0143 | 1.8E-09 | 0.0158 | | 1.9E-12 | |
| 9 | 129465325 | rs867559 | A | G | -0.0113 | 1.0E-04 | -0.015 | | 2.9E-08 | |
| 10 | 16299951 | rs10508503 | T | C | -0.0025 | 5.9E-01 | -0.0121 | | 4.1E-03 | |
| 10 | 87410904 | rs7899106 | A | G | -0.0258 | 1.9E-06 | -0.0375 | | 7.6E-14 | |
| 10 | 102395440 | rs17094222 | T | C | -0.0255 | 1.1E-18 | -0.0128 | | 1.9E-06 | |
| 10 | 104869038 | rs11191560 | T | C | -0.0349 | 1.3E-16 | -0.0193 | | 1.0E-06 | |
| 11 | 8604593 | rs4929949 | T | C | -0.0114 | 1.3E-06 | -0.0183 | | 1.2E-16 | |
| 11 | 8673939 | rs4256980 | C | G | -0.0143 | 5.1E-09 | -0.0221 | | 3.5E-22 | |
| 11 | 27725986 | rs10767664 | A | T | 0.0436 | 5.1E-49 | 0.0358 | | 2.0E-38 | |
| 11 | 43864278 | rs2176598 | T | C | 0.0204 | 5.4E-14 | 0.0202 | | 8.4E-16 | |
| 11 | 47650993 | rs3817334 | T | C | 0.0274 | 9.9E-31 | 0.0245 | | 3.0E-28 | |
| 11 | 115022404 | rs12286929 | A | G | -0.0178 | 2.3E-14 | -0.0178 | | 3.2E-16 | |
| 12 | 50247468 | rs7138803 | A | G | 0.0308 | 8.8E-37 | 0.0291 | | 9.7E-38 | |
| 12 | 122781897 | rs11057405 | A | G | -0.0276 | 4.1E-12 | -0.0313 | | 2.4E-17 | |
| 13 | 28017782 | rs9581854 | T | C | 0.0174 | 7.4E-08 | 0.0134 | | 6.4E-06 | |
| 13 | 28020180 | rs4771122 | A | G | -0.0147 | 8.0E-07 | -0.0103 | | 1.8E-04 | |
| 13 | 54102206 | rs12429545 | A | G | 0.0336 | 2.0E-21 | 0.0291 | | 1.1E-18 | |
| 14 | 25928179 | rs10132280 | A | C | -0.0299 | 1.4E-31 | -0.0153 | | 1.4E-10 | |
| 14 | 29736838 | rs12885454 | A | C | -0.0199 | 4.2E-16 | -0.018 | | 2.9E-15 | |
| 14 | 30515112 | rs11847697 | T | C | 0.0293 | 1.4E-07 | 0.0294 | | 9.3E-09 | |
| 14 | 79899454 | rs7141420 | T | C | 0.0213 | 1.2E-19 | 0.021 | | 7.8E-22 | |
| 14 | 79936964 | rs10150332 | T | C | -0.0285 | 2.7E-21 | -0.022 | | 2.2E-15 | |
| 15 | 51748610 | rs3736485 | A | G | 0.0108 | 5.2E-06 | 0.0159 | | 3.9E-13 | |
| 15 | 68077168 | rs16951275 | T | C | 0.0282 | 4.1E-24 | 0.0309 | | 8.7E-33 | |
| 15 | 68086838 | rs2241423 | A | G | -0.0285 | 1.5E-24 | -0.0308 | | 1.6E-32 | |
| 16 | 3627358 | rs758747 | T | C | 0.0161 | 1.3E-09 | 0.0121 | | 9.6E-07 | |
| 16 | 19933600 | rs12444979 | T | C | -0.0337 | 8.8E-23 | -0.0371 | | 6.4E-31 | |
| 16 | 19935389 | rs12446632 | A | G | -0.0338 | 4.3E-23 | -0.0375 | | 3.0E-32 | |
| 16 | 28333411 | rs2650492 | A | G | 0.0228 | 4.4E-18 | 0.0187 | | 1.1E-14 | |
| 16 | 28885659 | rs7359397 | T | C | 0.0305 | 1.4E-37 | 0.0259 | | 2.5E-31 | |
| 16 | 53803574 | rs1558902 | A | T | 0.0815 | 1.2E-257 | 0.0702 | | 1.1E-215 | |
| 16 | 53820527 | rs9939609 | A | T | 0.0785 | 4.4E-234 | 0.0672 | | 1.5E-199 | |
| 16 | 79682751 | rs1424233 | T | C | 0.0043 | 8.7E-02 | 0.0032 | | 1.6E-01 | |
| 17 | 5283252 | rs1000940 | A | G | -0.0165 | 1.5E-10 | -0.0159 | | 2.3E-11 | |
| 17 | 78615571 | rs12940622 | A | G | -0.0199 | 3.4E-17 | -0.0173 | | 5.0E-15 | |
| 18 | 21104888 | rs1808579 | T | C | -0.0201 | 1.7E-17 | -0.0198 | | 9.4E-20 | |
| 18 | 21140432 | rs1805081 | T | C | 0.0165 | 4.2E-12 | 0.0195 | | 1.3E-18 | |
| 18 | 56883319 | rs7243357 | T | G | 0.0249 | 5.3E-16 | 0.0153 | | 8.7E-08 | |
| 18 | 57829135 | rs6567160 | T | C | -0.0533 | 2.1E-82 | -0.0567 | | 8.7E-108 | |
| 18 | 57884750 | rs12970134 | A | G | 0.0467 | 3.4E-69 | 0.0508 | | 2.5E-94 | |
| 19 | 18454825 | rs17724992 | A | G | 0.0174 | 6.6E-11 | 0.017 | | 6.7E-12 | |
| 19 | 34309532 | rs29941 | A | G | -0.0162 | 6.5E-11 | -0.0153 | | 4.4E-11 | |
| 19 | 46202172 | rs2287019 | T | C | -0.0373 | 3.6E-33 | -0.0308 | | 1.5E-26 | |
| 19 | 47569003 | rs3810291 | A | G | 0.0286 | 1.4E-29 | 0.0251 | | 2.0E-26 | |

1. Yengo L, Sidorenko J, Kemper KE, Zheng Z, Wood AR, Weedon MN, et al. Meta-analysis of genome-wide association studies for height and body mass index in approximately 700000 individuals of European ancestry. Hum Mol Genet. 2018;27(20):3641-9.
