# Supplementary material for: Exclusive breastfeeding can attenuate body-mass-index increase among genetically susceptible children: A longitudinal study from the ALSPAC cohort
Source: PLoS Genet. 2020 Jun 11;16(6):e1008790. doi: 10.1371/journal.pgen.1008790 (PMC7289340; doi:10.1371/journal.pgen.1008790)
Supplement: S2 Table — (DOCX) [file pgen.1008790.s003.docx]

| AGE | Boys | | Girls | |
| --- | --- | --- | --- | --- |
|  | Effect (95% CI) | *p*-value | Effect (95% CI) | *p*-value |
| 0 | 0.01(-0.04,0.06) | 0.7862 | 0.00(-0.06,0.06) | 0.9137 |
| 1 | 0.01(-0.08,0.11) | 0.7792 | 0.03(-0.05,0.11) | 0.4751 |
| 2 | -0.04(-0.11,0.03) | 0.2952 | 0.03(-0.04,0.09) | 0.4147 |
| 3 | -0.05(-0.11,0.01) | 0.1108 | 0.04(-0.02,0.10) | 0.1738 |
| 4 | -0.02(-0.08,0.04) | 0.4826 | 0.08( 0.02,0.13) | 0.0080 |
| 5 | 0.04(-0.02,0.10) | 0.2223 | 0.14( 0.08,0.20) | <0.0001 |
| 6 | 0.12( 0.06,0.18) | 0.0001 | 0.21( 0.14,0.27) | <0.0001 |
| 7 | 0.22( 0.15,0.28) | <0.0001 | 0.29( 0.22,0.36) | <0.0001 |
| 8 | 0.31( 0.25,0.38) | <0.0001 | 0.37( 0.29,0.44) | <0.0001 |
| 9 | 0.41( 0.33,0.48) | <0.0001 | 0.45( 0.36,0.53) | <0.0001 |
| 10 | 0.48( 0.40,0.56) | <0.0001 | 0.51( 0.42,0.60) | <0.0001 |
| 11 | 0.53( 0.44,0.62) | <0.0001 | 0.56( 0.46,0.65) | <0.0001 |
| 12 | 0.57( 0.47,0.66) | <0.0001 | 0.59( 0.49,0.69) | <0.0001 |
| 13 | 0.59( 0.48,0.69) | <0.0001 | 0.61( 0.50,0.72) | <0.0001 |
| 14 | 0.60( 0.49,0.71) | <0.0001 | 0.62( 0.50,0.73) | <0.0001 |
| 15 | 0.62( 0.50,0.73) | <0.0001 | 0.61( 0.49,0.73) | <0.0001 |
| 16 | 0.64( 0.51,0.77) | <0.0001 | 0.60( 0.47,0.73) | <0.0001 |
| 17 | 0.68( 0.54,0.82) | <0.0001 | 0.58( 0.44,0.72) | <0.0001 |
| 18 | 0.74( 0.58,0.90) | <0.0001 | 0.55( 0.39,0.71) | <0.0001 |
