# Supplementary material for: Exclusive breastfeeding can attenuate body-mass-index increase among genetically susceptible children: A longitudinal study from the ALSPAC cohort
Source: PLoS Genet. 2020 Jun 11;16(6):e1008790. doi: 10.1371/journal.pgen.1008790 (PMC7289340; doi:10.1371/journal.pgen.1008790)
Supplement: S3 Table — The 95% confidence intervals (CIs) are computed with the bootstrap method3 with 2,000 iterations. (DOCX) [file pgen.1008790.s004.docx]

|  | Boys | | |  | Girls | |
| --- | --- | --- | --- | --- | --- | --- |
| GRS | **AP** | Effect (95%CI) | *p*-value | **AP** | Effect (95%CI) | *p*-value |
| 2.5 | 0.77 |  |  | 0.75 |  |  |
| 5 | 0.79 | 0.02 (-0.05, 0.07) | 0.7121 | 0.78 | 0.03 (-0.01, 0.06) | 0.1593 |
| 7.5 | 0.80 | 0.03 (-0.09, 0.13) | 0.7334 | 0.80 | 0.05 (-0.02, 0.0.12) | 0.1381 |
|  |  |  |  |  |  |  |
| GRS | **AR** | Effect (95%CI) | *p*-value | **AR** | Effect (95%CI) | *p*-value |
| 2.5 | 5.43 |  |  | 5.09 |  |  |
| 5 | 5.07 | -0.36 (-0.46, -0.27) | <0.0001 | 4.78 | -0.31 (-0.41, -0.21) | <0.0001 |
| 7.5 | 4.78 | -0.65 (-0.80, -0.49) | <0.0001 | 4.52 | -0.57 (-0.74, -0.39) | <0.0001 |
