# Supplementary material for: Exclusive breastfeeding can attenuate body-mass-index increase among genetically susceptible children: A longitudinal study from the ALSPAC cohort
Source: PLoS Genet. 2020 Jun 11;16(6):e1008790. doi: 10.1371/journal.pgen.1008790 (PMC7289340; doi:10.1371/journal.pgen.1008790)
Supplement: S4 Table — The optimal knots were (K1=0.7, K2=1.5, K3=10) for boys and (K1=0.9, K2=1.5, K3=10) for girls. The knots were defined in supplementary file section A.2. (DOCX) [file pgen.1008790.s005.docx]

|  | Boys | | | Girls | | | | | |  |
| --- | --- | --- | --- | --- | --- | --- | --- | --- | --- | --- |
|  | BETA | SE | p-value | BETA | | SE | | p-value | |  |
| *sp*(*A*$ge$)*GRS*EBF | -0.1239 | 0.0663 | **0.0619** | -0.2117 | | 0.0815 | | **0.0094** | |  |
| *sp*(${Age}^{2}$)*GRS*EBF | 0.4914 | 0.2881 | **0.0881** | 0.7102 | | 0.2910 | | **0.0147** | |  |
| *sp*(${Age}^{3}$)*GRS*EBF | -0.7891 | 0.4887 | **0.1064** | -0.9526 | | 0.4027 | | **0.0180** | |  |
| *sp*(${Age}_{\mathcal{K}_{1}}^{3}$)*GRS*EBF | 0.8640 | 0.5599 | 0.1228 | 1.1994 | | 0.5257 | | **0.0225** | |  |
| *sp*(${Age}_{\mathcal{K}_{2}}^{3}$)*GRS*EBF | -0.0747 | 0.0792 | 0.3455 | -0.2470 | | 0.1277 | | **0.0530** | |  |
| *sp*(${Age}_{\mathcal{K}_{3}}^{3}$)*GRS*EBF | -0.0006 | 0.0008 | 0.4966 | 0.0004 | | 0.0006 | | 0.4702 | |  |
|  | | | | |  | |  | |  | |
|  | Boys | | | Girls | | | | | |  |
|  | BETA | SE | *p*-value | BETA | | SE | | *p*-value | |  |
| *sp*(*A*$ge$)*GRS*BF | -0.0813 | 0.0222 | **0.0003** | -0.0539 | | 0.0264 | | **0.0410** | |  |
| *sp*(${Age}^{2}$)*GRS*BF | 0.3290 | 0.0967 | **0.0007** | 0.1846 | | 0.0941 | | **0.0497** | |  |
| *sp*(${Age}^{3}$)*GRS*BF | -0.5329 | 0.1640 | **0.0012** | -0.2470 | | 0.1302 | | **0.0578** | |  |
| *sp*(${Age}_{\mathcal{K}_{1}}^{3}$)*GRS*BF | 0.5879 | 0.1879 | **0.0018** | 0.3096 | | 0.1700 | | **0.0686** | |  |
| *sp*(${Age}_{\mathcal{K}_{2}}^{3}$)*GRS*BF | -0.0550 | 0.0265 | **0.0383** | -0.0626 | | 0.0414 | | 0.1306 | |  |
| *sp*(${Age}_{\mathcal{K}_{3}}^{3}$)*GRS*EBF | -0.0001 | 0.0003 | 0.8371 | -0.0001 | | 0.0002 | | 0.6756 | |  |
