# Supplementary material for: Exclusive breastfeeding can attenuate body-mass-index increase among genetically susceptible children: A longitudinal study from the ALSPAC cohort
Source: PLoS Genet. 2020 Jun 11;16(6):e1008790. doi: 10.1371/journal.pgen.1008790 (PMC7289340; doi:10.1371/journal.pgen.1008790)
Supplement: S5 Table — (DOCX) [file pgen.1008790.s006.docx]

| AGE | GRS | EBF/BF Duration |  | 5 months EBF Effect | |  | 5 months BF Effect | |  |  |
| --- | --- | --- | --- | --- | --- | --- | --- | --- | --- | --- |
|  |  |  | BMI | Effect (95% CI) | *p*-value | BMI | Effect (95% CI) | *p*-value | |  |
| ***ALSPAC Boys*** | | | | | | | | | | |
| 7 | 2.5 | 0 | 15.92 |  |  | 15.97 |  |  | |  |
|  |  | 5 | 15.71 | -0.21 (-0.53, 0.10) | 0.1876 | 15.85 | -0.12 (-0.22,-0.01) | 0.0266 | |  |
|  | 5 | 0 | 16.11 |  |  | 16.14 |  |  | |  |
|  |  | 5 | 15.99 | -0.12 (-0.37, 0.12) | 0.3238 | 16.07 | -0.07 (-0.15, 0.01) | 0.0947 | |  |
|  | 7.5 | 0 | 16.30 |  |  | 16.30 |  |  | |  |
|  |  | 5 | 16.26 | -0.03 (-0.35, 0.29) | 0.848 | 16.28 | -0.02 (-0.13, 0.09) | 0.7127 | |  |
| 10 | 2.5 | 0 | 17.29 |  |  | 17.34 |  |  | |  |
|  |  | 5 | 16.87 | -0.43 (-0.83,-0.02) | 0.0387 | 17.15 | -0.19 (-0.32,-0.06) | 0.0051 | |  |
|  | 5 | 0 | 17.75 |  |  | 17.76 |  |  | |  |
|  |  | 5 | 17.38 | -0.37 (-0.68,-0.07) | 0.0176 | 17.63 | -0.13 (-0.23,-0.03) | 0.0123 | |  |
|  | 7.5 | 0 | 18.21 |  |  | 18.18 |  |  | |  |
|  |  | 5 | 17.89 | -0.32 (-0.73, 0.09) | 0.1225 | 18.11 | -0.07 (-0.21, 0.06) | 0.2971 | |  |
| 15 | 2.5 | 0 | 20.39 |  |  | 20.38 |  |  | |  |
|  |  | 5 | 19.77 | -0.61 (-1.19,-0.03) | 0.0378 | 20.18 | -0.21 (-0.40,-0.02) | 0.0335 | |  |
|  | 5 | 0 | 20.99 |  |  | 20.96 |  |  | |  |
|  |  | 5 | 20.39 | -0.60 (-1.04,-0.16) | 0.0077 | 20.79 | -0.17 (-0.32,-0.03) | 0.0215 | |  |
|  | 7.5 | 0 | 21.60 |  |  | 21.54 |  |  | |  |
|  |  | 5 | 21.01 | -0.58 (-1.16, 0.00) | 0.0496 | 21.40 | -0.14 (-0.33, 0.06) | 0.169 | |  |
| 18 | 2.5 | 0 | 22.38 |  |  | 22.42 |  |  | |  |
|  |  | 5 | 21.57 | -0.81 (-1.57,-0.05) | 0.0362 | 22.11 | -0.31 (-0.56,-0.05) | 0.0172 | |  |
|  | 5 | 0 | 23.17 |  |  | 23.18 |  |  | |  |
|  |  | 5 | 22.19 | -0.98 (-1.56,-0.40) | 0.001 | 22.84 | -0.34 (-0.54,-0.15) | 0.0005 | |  |
|  | 7.5 | 0 | 23.95 |  |  | 23.95 |  |  | |  |
|  |  | 5 | 22.81 | -1.14 (-1.91,-0.37) | 0.0037 | 23.57 | -0.37 (-0.63,-0.12) | 0.0042 | |  |
| ***ALSPAC Girls*** | | | | | | | | | | |
| 7 | 2.5 | 0 | 16.03 |  |  | 16.06 |  |  | |  |
|  |  | 5 | 15.65 | -0.38 (-0.72,-0.04) | 0.0272 | 15.90 | -0.16 (-0.27,-0.04) | 0.0068 | |  |
|  | 5 | 0 | 16.36 |  |  | 16.31 |  |  | |  |
|  |  | 5 | 15.86 | -0.50 (-0.76,-0.24) | 0.0002 | 16.19 | -0.12 (-0.21,-0.04) | 0.0061 | |  |
|  | 7.5 | 0 | 16.70 |  |  | 16.57 |  |  | |  |
|  |  | 5 | 16.07 | -0.62 (-0.96,-0.28) | 0.0003 | 16.48 | -0.09 (-0.21, 0.02) | 0.1216 | |  |
| 10 | 2.5 | 0 | 17.71 |  |  | 17.76 |  |  | |  |
|  |  | 5 | 16.91 | -0.80 (-1.23,-0.38) | 0.0002 | 17.44 | -0.32 (-0.47,-0.18) | <0.0001 | |  |
|  | 5 | 0 | 18.27 |  |  | 18.24 |  |  | |  |
|  |  | 5 | 17.33 | -0.95 (-1.27,-0.62) | <0.0001 | 17.94 | -0.30 (-0.41,-0.18) | <0.0001 | |  |
|  | 7.5 | 0 | 18.83 |  |  | 18.72 |  |  | |  |
|  |  | 5 | 17.74 | -1.09 (-1.52,-0.66) | <0.0001 | 18.45 | -0.27 (-0.41,-0.12) | 0.0004 | |  |
| 15 | 2.5 | 0 | 21.27 |  |  | 21.30 |  |  | |  |
|  |  | 5 | 20.33 | -0.93 (-1.51,-0.35) | 0.0017 | 20.94 | -0.35 (-0.55,-0.16) | 0.0004 | |  |
|  | 5 | 0 | 21.98 |  |  | 21.92 |  |  | |  |
|  |  | 5 | 20.76 | -1.22 (-1.66,-0.77) | <0.0001 | 21.55 | -0.37 (-0.52,-0.21) | <0.0001 | |  |
|  | 7.5 | 0 | 22.69 |  |  | 22.54 |  |  | |  |
|  |  | 5 | 21.19 | -1.51 (-2.09,-0.92) | <0.0001 | 22.17 | -0.38 (-0.58,-0.17) | 0.0003 | |  |
| 18 | 2.5 | 0 | 22.75 |  |  | 22.79 |  |  | |  |
|  |  | 5 | 21.89 | -0.86 (-1.62,-0.11) | 0.0252 | 22.45 | -0.34 (-0.60,-0.09) | 0.0075 | |  |
|  | 5 | 0 | 23.42 |  |  | 23.45 |  |  | |  |
|  |  | 5 | 22.23 | -1.20 (-1.77,-0.62) | <0.0001 | 23.01 | -0.44 (-0.64,-0.24) | <0.0001 | |  |
|  | 7.5 | 0 | 24.09 |  |  | 24.11 |  |  | |  |
|  |  | 5 | 22.57 | -1.53 (-2.29,-0.76) | <0.0001 | 23.57 | -0.54 (-0.81,-0.27) | <0.0001 | |  |
