# Supplementary material for: Exclusive breastfeeding can attenuate body-mass-index increase among genetically susceptible children: A longitudinal study from the ALSPAC cohort
Source: PLoS Genet. 2020 Jun 11;16(6):e1008790. doi: 10.1371/journal.pgen.1008790 (PMC7289340; doi:10.1371/journal.pgen.1008790)
Supplement: S6 Table — The 95% confidence intervals (CIs) are computed with the bootstrap method with 2,000 iterations. (DOCX) [file pgen.1008790.s007.docx]

|  |  | Boys | | | Girls | | |
| --- | --- | --- | --- | --- | --- | --- | --- |
| GRS | EBF | **AP** | Effect (95%CI) | *p*-value | **AP** | Effect (95%CI) | *p*-value |
| 2.5 | 0 | 0.71 |  |  | 0.76 |  |  |
|  | 3 | 0.82 | 0.11 (-0.05,0.25) | 0.1815 | 0.75 | -0.01 (-0.09,0.08) | 0.9075 |
|  | 5 | 0.88 | 0.17 (-0.07,0.37) | 0.1712 | 0.75 | -0.01 (-0.15,0.14) | 0.9485 |
| 5 | 0 | 0.71 |  |  | 0.75 |  |  |
|  | 3 | 0.85 | 0.14 (0.03,0.25) | 0.0158 | 0.81 | 0.06 (-0.01,0.14) | 0.1021 |
|  | 5 | 0.92 | 0.21 (0.05,0.36) | 0.0076 | 0.87 | 0.12 (-0.02,0.26) | 0.0951 |
| 7.5 | 0 | 0.70 |  |  | 0.74 |  |  |
|  | 3 | 0.87 | 0.17 (0.02,0.30) | 0.0246 | 0.88 | 0.14 (0.04,0.24) | 0.0063 |
|  | 5 | 0.95 | 0.25 (0.05,0.42) | 0.0136 | 0.98 | 0.24 (0.09,0.38) | 0.0011 |
|  |  | Boys | | | Girls | | |
| GRS | EBF | **AR** | Effect (95%CI) | *p*-value | **AR** | Effect (95%CI) | *p*-value |
| 2.5 | 0 | 5.36 |  |  | 4.90 |  |  |
|  | 3 | 5.48 | 0.12 (-0.12,0.34) | 0.3434 | 5.25 | 0.35 (0.08,0.63) | 0.0105 |
|  | 5 | 5.57 | 0.19 (-0.21,0.61) | 0.3434 | 5.54 | 0.64 (0.15,1.16) | 0.0114 |
| 5 | 0 | 5.01 |  |  | 4.62 |  |  |
|  | 3 | 5.11 | 0.10 (-0.07,0.27) | 0.268 | 4.91 | 0.29 (0.11,0.47) | 0.0013 |
|  | 5 | 5.19 | 0.18 (-0.13,0.48) | 0.2672 | 5.15 | 0.53 (0.20,0.86) | 0.0015 |
| 7.5 | 0 | 4.74 |  |  | 4.39 |  |  |
|  | 3 | 4.82 | 0.08 (-0.13,0.29) | 0.4626 | 4.63 | 0.24 (0.03,0.47) | 0.0262 |
|  | 5 | 4.88 | 0.14 (-0.24,0.53) | 0.4578 | 4.83 | 0.44 (0.05,0.85) | 0.0278 |
