# Supplementary material for: Exclusive breastfeeding can attenuate body-mass-index increase among genetically susceptible children: A longitudinal study from the ALSPAC cohort
Source: PLoS Genet. 2020 Jun 11;16(6):e1008790. doi: 10.1371/journal.pgen.1008790 (PMC7289340; doi:10.1371/journal.pgen.1008790)
Supplement: S7 Table — (DOCX) [file pgen.1008790.s008.docx]

| AGE | GRS | EBF/BF Duration |  | 3 months EBF Effect | |  | 3 months BF Effect | |  |
| --- | --- | --- | --- | --- | --- | --- | --- | --- | --- |
|  |  |  | BMI | Effect (95% CI) | *p*-value | BMI | Effect (95% CI) | *p*-value |  |
| ***ALSPAC Boys*** | | | | | | | | | |
| 7 | 2.5 | 0 | 15.92 |  |  | 15.97 |  |  |  |
|  |  | 3 | 15.80 | -0.13 (-0.32, 0.06) | 0.1876 | 15.90 | -0.07 (-0.13,-0.01) | 0.0266 |  |
|  | 5 | 0 | 16.11 |  |  | 16.14 |  |  |  |
|  |  | 3 | 16.04 | -0.07 (-0.22, 0.07) | 0.3238 | 16.10 | -0.04 (-0.09, 0.01) | 0.0947 |  |
|  | 7.5 | 0 | 16.30 |  |  | 16.30 |  |  |  |
|  |  | 3 | 16.28 | -0.02 (-0.21, 0.17) | 0.848 | 16.29 | -0.01 (-0.08, 0.05) | 0.7127 |  |
| 10 | 2.5 | 0 | 17.29 |  |  | 17.34 |  |  |  |
|  |  | 3 | 17.04 | -0.26 (-0.50,-0.01) | 0.0387 | 17.22 | -0.11 (-0.19,-0.03) | 0.0051 |  |
|  | 5 | 0 | 17.75 |  |  | 17.76 |  |  |  |
|  |  | 3 | 17.53 | -0.22 (-0.41,-0.04) | 0.0176 | 17.68 | -0.08 (-0.14,-0.02) | 0.0123 |  |
|  | 7.5 | 0 | 18.21 |  |  | 18.18 |  |  |  |
|  |  | 3 | 18.02 | -0.19 (-0.44, 0.05) | 0.1225 | 18.13 | -0.04 (-0.12, 0.04) | 0.2971 |  |
| 15 | 2.5 | 0 | 20.39 |  |  | 20.38 |  |  |  |
|  |  | 3 | 20.02 | -0.37 (-0.71,-0.02) | 0.0378 | 20.26 | -0.12 (-0.24,-0.01) | 0.0335 |  |
|  | 5 | 0 | 20.99 |  |  | 20.96 |  |  |  |
|  |  | 3 | 20.63 | -0.36 (-0.62,-0.09) | 0.0077 | 20.86 | -0.10 (-0.19,-0.02) | 0.0215 |  |
|  | 7.5 | 0 | 21.60 |  |  | 21.54 |  |  |  |
|  |  | 3 | 21.25 | -0.35 (-0.70, 0.00) | 0.0496 | 21.46 | -0.08 (-0.20, 0.03) | 0.169 |  |
| 18 | 2.5 | 0 | 22.38 |  |  | 22.42 |  |  |  |
|  |  | 3 | 21.89 | -0.49 (-0.94,-0.03) | 0.0362 | 22.23 | -0.19 (-0.34,-0.03) | 0.0172 |  |
|  | 5 | 0 | 23.17 |  |  | 23.18 |  |  |  |
|  |  | 3 | 22.58 | -0.59 (-0.93,-0.24) | 0.001 | 22.98 | -0.21 (-0.32,-0.09) | 0.0005 |  |
|  | 7.5 | 0 | 23.95 |  |  | 23.95 |  |  |  |
|  |  | 3 | 23.27 | -0.68 (-1.14,-0.22) | 0.0037 | 23.72 | -0.22 (-0.38,-0.07) | 0.0042 |  |
| ***ALSPAC Girls*** | | | | | | | | | |
| 7 | 2.5 | 0 | 16.03 |  |  | 16.06 |  |  |  |
|  |  | 3 | 15.81 | -0.23(-0.43,-0.03) | 0.0272 | 15.96 | -0.09 (-0.16,-0.03) | 0.0068 |  |
|  | 5 | 0 | 16.36 |  |  | 16.31 |  |  |  |
|  |  | 3 | 16.06 | -0.30(-0.46,-0.15) | 0.0002 | 16.24 | -0.07 (-0.13,-0.02) | 0.0061 |  |
|  | 7.5 | 0 | 16.70 |  |  | 16.57 |  |  |  |
|  |  | 3 | 16.32 | -0.37(-0.58,-0.17) | 0.0003 | 16.51 | -0.06 (-0.13, 0.01) | 0.1216 |  |
| 10 | 2.5 | 0 | 17.71 |  |  | 17.76 |  |  |  |
|  |  | 3 | 17.23 | -0.48(-0.74,-0.23) | 0.0002 | 17.57 | -0.19 (-0.28,-0.11) | <0.0001 |  |
|  | 5 | 0 | 18.27 |  |  | 18.24 |  |  |  |
|  |  | 3 | 17.71 | -0.57(-0.76,-0.37) | <0.0001 | 18.06 | -0.18 (-0.24,-0.11) | <0.0001 |  |
|  | 7.5 | 0 | 18.83 |  |  | 18.72 |  |  |  |
|  |  | 3 | 18.18 | -0.65(-0.91,-0.40) | <0.0001 | 18.56 | -0.16 (-0.25,-0.07) | 0.0004 |  |
| 15 | 2.5 | 0 | 21.27 |  |  | 21.30 |  |  |  |
|  |  | 3 | 20.71 | -0.56(-0.91,-0.21) | 0.0017 | 21.08 | -0.21 (-0.33,-0.09) | 0.0004 |  |
|  | 5 | 0 | 21.98 |  |  | 21.92 |  |  |  |
|  |  | 3 | 21.25 | -0.73(-1.00,-0.46) | <0.0001 | 21.70 | -0.22 (-0.31,-0.13) | <0.0001 |  |
|  | 7.5 | 0 | 22.69 |  |  | 22.54 |  |  |  |
|  |  | 3 | 21.79 | -0.90(-1.26,-0.55) | <0.0001 | 22.32 | -0.23 (-0.35,-0.10) | 0.0003 |  |
| 18 | 2.5 | 0 | 22.75 |  |  | 22.79 |  |  |  |
|  |  | 3 | 22.23 | -0.52(-0.97,-0.06) | 0.0252 | 22.58 | -0.21 (-0.36,-0.06) | 0.0075 |  |
|  | 5 | 0 | 23.42 |  |  | 23.45 |  |  |  |
|  |  | 3 | 22.71 | -0.72(-1.06,-0.37) | <0.0001 | 23.18 | -0.27 (-0.38,-0.15) | <0.0001 |  |
|  | 7.5 | 0 | 24.09 |  |  | 24.11 |  |  |  |
|  |  | 3 | 23.18 | -0.92(-1.38,-0.46) | <0.0001 | 23.78 | -0.32 (-0.48,-0.16) | <0.0001 |  |
