# Supplementary material for: Exclusive breastfeeding can attenuate body-mass-index increase among genetically susceptible children: A longitudinal study from the ALSPAC cohort
Source: PLoS Genet. 2020 Jun 11;16(6):e1008790. doi: 10.1371/journal.pgen.1008790 (PMC7289340; doi:10.1371/journal.pgen.1008790)
Supplement: Attachment — Submitted filename: ReviewerComments_March25_a.docx [file pgen.1008790.s010.docx]

Wednesday, March 25, 2020

We thank the associate editor and the three reviewers for their very thorough review of our MS. Their comments have been helpful to improve our MS. We provide below a point-by-point reply to their questions.

**Associate editor:**

There are multiple concerns raised by the Reviewers needs to be addressed and that prevent publication of the manuscript in its current form, in particular:

1) Please clarify what is the novelty of the current manuscript compared to the previous papers from ALSPAC and other data,

We have clarified the novelty in the introduction of the MS.

2) Please clarify the potential overlap of samples between the PRS weights and the test cohort and use other source for weights if they exist,

We have upated the weights based on an independent external study.

and 3) present a formal statistical test for interaction as the basis for inference.

We explained how the formal tests were performed for the interaction. (See our response to Q4 from the first reviewer)

Reviewer's Responses to Questions

**Comments to the Authors:
Please note here if the review is uploaded as an attachment.**

Reviewer #1: Thank you for the opportunity to review an interesting manuscript from effects of exclusive of breast feeding (EBF) and genetics on BMI trajectories. The Results section is clearly written and describes the age- and sex dependent complex relationships very well. However, there are some points which require clarification, especially the sample used to derive the SNP weights.

1) What sample was used to derive the SNP weights for the GRS? Was there overlap with ALSPAC?

We initially used the same sample to derive the SNP weights but in reply to the reviewers’ comments, we have now recalculated these weights of all the 94 SNPs forming the GRS using the sex-specific weights derived from the GIANT consortium and UK BiobBank Meta-analysis (Yengo et al., 2018) and available through the portal: <https://portals.broadinstitute.org/collaboration/giant/index.php/GIANT_consortium_data_files>.

We edited the text on page 7 line 144-148:

The sex-specific GRSs were created using the imputed dosages for the 94 SNPs where each SNP was recoded to represent the number of BMI-increasing alleles and was weighted using the sex-specific weights derived from the GIANT consortium and UK BiobBank meta-analysis [19] and available through the portal: https://portals.broadinstitute.org/collaboration/giant/index.php/GIANT_consortium_data_files.

2) Increased maternal BMI is a risk factor for emergency caesarean section and mode of delivery is associated for example with long-term changes in gut microbiota compared to vaginal delivery. As approximately half of the GRS is inherited from mother and thus affects also maternal BMI, could mode of delivery be a confounding factor?

We did not adjust our analyses for the mode of delivery but we did adjust them for mother's pre-pregnancy BMI.

3) In the discussion, you suggest that obesity interventions could target subjects in the upper tail of GRS. What GRS percentile you would target? Could any intervention be effective enough to justify screening costs considering that by targeting upper tail most of the obesity cases are missed (in absolute numbers due to Rose paradox)? Why do you think that targeted intervention would be more reasonable approach than non-targeted one?

We have added the following paragraph to the discussion:

‘Recent efforts have demonstrated the clinical utility of the GRS in predicting overweight and obesity risks [13, 14]. A recent paper using an extended version of the GRS based on 2.1 million genetic variants, stressed the greatly increased risk of severe obesity among individuals in the top decile of the GRS. For instance, 15.6% of individuals in the top decile of GRS went on to develop severe obesity compared with 5.6% of those in deciles 2-9 and 1.3% in the lowest decile [13]. This top decile of the population had also a 4.2, 6.6 and 14.4-fold increased risk of a high BMI of 40, 50 and 60 compared to the rest of the population and also had increased risks of cardiometabolic diseases and overall mortality. Targeting this 10% decile population might therefore offer a cost-efficient strategy to reduce obesity-related morbidity, although this would need to be thoroughly evaluated. These authors also stressed the importance of early intervention, acknowledging that “given that the weight trajectories of individuals in different GRS deciles start to diverge in early childhood, such interventions may have maximal effect when employed early in life.” As noted also in Torkamani et. al. [14], a targeted intervention might help “clarifying a high-risk individual’s perception of their susceptibility to disease and quantifying the benefits of healthy behaviors could be an effective tool to induce and maintain behavioral changes”.’

4) Table 3: In boys, the effect of EBF on BMI seems to be larger when GRS is high. In girls, it seems to be the opposite (for example in 15 yo -1.55 if GRS=2.5 and -0.87 if GRS=7.5). Any suggestions why?

Using the new weights from Yengo L et.al 2018, we have now the same trend in boys and girls, where the effect of EBF on BMI is larger for high values of the GRS in both boys and girls. For instance, at 18 years of age, EBF to 5 months reduces BMI by 1.14 and 1.53 at the third quartile of GRS in boys and girls, respectively, but only by 0.81 and 0.86, at the first quartile of GRS.

Reviewer #2: The authors have performed a study on the association of exclusive breastfeeding with BMI, adiposity peak, and adiposity rebound in 5,266 children from the ALSPAC cohort, and the interaction of exclusive breastfeeding with a genetic risk score for increased BMI. The manuscript reports that exclusive breastfeeding reduces BMI in adolescents and attenuates the impact of a genetic risk score for BMI. While the results are interesting, the novelty of the study seems rather limited. The authors have previously published results from a similar analysis of the interaction between exclusive breastfeeding and the FTO obesity risk locus on BMI trajectories in the ALSPAC cohort [29040503]. The link between breastfeeding and child BMI has been previously studied in ALSPAC, by Brion et al. [PMID 21349903], although in somewhat less detail than here.

The reviewer is correct that we have already investigated the role of EBF in mitigating the effect of a FTO genetic variant on BMI in children, as acknowledged in the introduction of the MS. To better justify this new contribution, we have added the following paragraph at the end of the introduction:

“While knowledge on the genetic architecture of adult and pediatric BMI is accumulating thanks to large-scale GWAS results, the construction of obesity-specific GRSs is emerging as an important approach for the personal and clinical management of individuals at risk of adverse outcomes (Tokomani et al., 2018; Khera et al., 2019). It is therefore timely to consider the protective effect of EBF among children with elevated risk of overweight/obesity, where this risk is assessed by an obesity-specific GRS, and thus to extend our previous results on the *FTO* genetic variant. Our goal in this paper is to assess the effect of this GRS from infancy to the end of adolescence as well as the modulating effect of EBF during this time period.”

My other main comments are the following:

1. The authors have used a weighted GRS, but the way the variants have been weighted when constructing the GRS is worrying. If I understand correctly, the effect sizes used for weighting were obtained from analyses of the ALSPAC dataset, i.e. the same dataset that was utilized in the present analyses. This leads to a bias where the effect of the GRS on BMI is inflated. To construct the GRS correctly, the weights would need to be extracted from the original GWAS discovery study or another large, independent dataset. Alternatively, an unweighted GRS could be used.

Thanks for the suggestion. We initially used the same sample to derive the SNP weights but now, we have recalculated these weights for all the 94 SNPs forming the GRS using sex-specific weights derived from the GIANT consortium and UK BiobBank meta-analysis (Yengo et al., 2018) and available through the portal: [https://portals.broadinstitute.org/collaboration/giant/index.php/ GIANT_consortium_data_files](https://portals.broadinstitute.org/collaboration/giant/index.php/%20GIANT_consortium_data_files).

2. The authors have combined results from 97 independent variants only, rather than taking advantage of more recent latest GWAS including >400 independent variants [PMID 30239722].

We have not included this GRS definition because the research on BMI-related and obesity-related GRSs is moving quickly and new definitions such as the 941-SNPs of Yengo et.al. (2018) and the 2.1 million-SNPs GRS of Khera et al. (2019) have just been released. It is likely that these emerging definitions of GRSs will keep updating and could soon integrate as well genetic variants identified through large meta-GWASs conducted specifically on children and/or estimate weights of GRSs specifically from child populations. We added the following paragraph to the discussion section:

We have added the following sentence to the discussion:

“Our GRS definition was based on 94 SNPs including 69 SNPs from Locke et.al., 2015 [6]. As large meta-GWASs on BMI and obesity-related traits are fast developing, some extended definitions of GRSs are emerging such as Yengo et.al. 2018 [19] and Khera et.al, 2019 [13]. However, the correlation between BMI and GRS in the Health and Retirement Study participants derived from Locke at.al., 2015 is near identical to the correlation between the BMI and GRS derived from Yengo et.al., 2018 (r = 0.22) [32]. We are planning to generalize our study to these new GRS definitions in the near future. Also, our GRS definition is mainly based on SNPs found associated with BMI in adults and could be extended to include genetic variants more specific to children, taking advantage on recent GWAS discoveries [33-35].”

3. The authors state that “the effect of the GRS is not well studied in children” (p. 3) and that “the role of the obesity-specific GRS remains largely unknown in children and adolescents” (p. 15). However, many studies of obesity-specific GRS in children have been published [e.g. PMID 29211904, 30515969, 28008729, 24244521]. Thus, these claims do not seem valid.

We changed the 2 bullets on in the section "what is already known on this topic" on p3:

- A GRS based on 97 genetic variants has been derived from large GWASs and is predictive of BMI in adults and children.
- It remains unclear whether EBF can attenuate the increase in BMI attributed to the GRS in children.

We also changed the sentence on p15 to: " The role of the obesity-specific GRS has been recently studied in children and adolescents [10-13, 23-28] and recent evidence suggests" and cited these references.

13.	Khera AV, Chaffin M, Wade KH, Zahid S, Brancale J, Xia R, et al. Polygenic Prediction of Weight and Obesity Trajectories from Birth to Adulthood. Cell. 2019;177(3):587-96.e9.

23.	Viitasalo A, Schnurr TM, Pitkanen N, Hollensted M, Nielsen TRH, Pahkala K, et al. Genetic predisposition to higher body fat yet lower cardiometabolic risk in children and adolescents. Int J Obes (Lond). 2019;43(10):2007-16.

24.	Justice AE, Chittoor G, Blanco E, Graff M, Wang Y, Albala C, et al. Genetic determinants of BMI from early childhood to adolescence: the Santiago Longitudinal Study. Pediatr Obes. 2019;14(3):e12479.

25.	Li A, Robiou-du-Pont S, Anand SS, Morrison KM, McDonald SD, Atkinson SA, et al. Parental and child genetic contributions to obesity traits in early life based on 83 loci validated in adults: the FAMILY study. Pediatr Obes. 2018;13(3):133-40.

26.	Steinsbekk S, Belsky D, Guzey IC, Wardle J, Wichstrom L. Polygenic Risk, Appetite Traits, and Weight Gain in Middle Childhood: A Longitudinal Study. JAMA Pediatr. 2016;170(2):e154472.

27.	Elks CE, Heude B, de Zegher F, Barton SJ, Clement K, Inskip HM, et al. Associations between genetic obesity susceptibility and early postnatal fat and lean mass: an individual participant meta-analysis. JAMA Pediatr. 2014;168(12):1122-30.

28.	Belsky DW, Moffitt TE, Houts R, Bennett GG, Biddle AK, Blumenthal JA, et al. Polygenic risk, rapid childhood growth, and the development of obesity: evidence from a 4-decade longitudinal study. Arch Pediatr Adolesc Med. 2012;166(6):515-21.

4. The authors report having evaluated the interaction between the duration of exclusive breastfeeding and GRS on BMI growth trajectories. However, they have not performed a formal test for interaction, but rather base their conclusions on observing values between stratified subgroups, which does not seem sufficient statistical evidence for an interaction. The authors should perform a formal test for interaction by including an interaction term in the model. Similar issue applies to e.g. p. 14 where the authors state that “a duration of EBF for 3 months had significantly less impact in decreasing BMI than 5 months of EBF”. A formal test for the difference between the groups should be included here, to make such claim about statistical significance.

We actually did not previously performed a test of GRS*EBF interaction by age-specific subgroups but instead estimated this effect at different time-points from the whole cohort, separately for boys and girls. This is possible by using the generalized linear hypothesis approach (GLH, Appendix A2). It is a Wald test, a likelihood-based method, which tests formally or compares differences by specifying age, GRS value, EBF (or BF) value and other covariates through a constant matrix or vector *L* to be matched with the parameter estimates of the model ($\hat{\beta})$. The parameter estimates follow asymptotically a multivariate normal distribution $\hat{\beta}\sim N(\beta, cov\left( \hat{\beta} \right))$ from Central Limit Theorem such that the linear form $L\hat{\beta}$also follows asymptotically a multivariate normal distribution: $L\hat{\beta}\sim N\left( L\beta, Lcov\left( \hat{\beta} \right)L^{'} \right).$ Therefore the *p*-value and the 95% confidence interval for the hypothesized value can be obtained accordingly. For example, to estimate and to test a 5-months EBF effect at age 10 for boys, the *L* matrix is$L\hat{\beta}=L_{2}^{'}\hat{\beta}-L_{1}^{'}\hat{\beta}$ where $L_{2}^{'}\hat{\beta}$ is the estimated mean BMI assuming age = 10 and EBF = 5, while $L_{1}^{'}\hat{\beta}$ assumes age=10 and EBF = 0, holding all other covariates constant.

Based on this approach, we can assess that the test of the 3-way interaction is significant after 10 years of age for certain categories of GRS (table S5), which is more relevant than a “global” test over all ages since the GRS itself has a significant effect on BMI only from 5 and 6 years onwards in girls and boys, respectively (previous table S2).

To answer the reviewer’s question, we however provide in Appendix table S2, a specific test of significance for the different components of the 3-way interaction for both EBF and BF. It shows that the main component *sp*(A$ge$)*GRS*EBF which measures a linear time trend is always highly significant except for boys and EBF, where the test is borderline significant, i.e., p=0.062.

Table S2. Three-way interaction of cubic splines of age, GRS and breastfeeding (EBF: exclusive breastfeeding, BF: non-exclusive breastfeeding) for boys and girls. The optimal knots were ($\mathcal{K}_{1}$=0.7, $\mathcal{K}_{2}$=1.5, $\mathcal{K}_{3}$=10) for boys and ($\mathcal{K}_{1}$=0.9, $\mathcal{K}_{2}$=1.5, $\mathcal{K}_{3}$=10) for girls. The knots were defined in supplementary file section A.2.

|  | Boys | | | Girls | | | | | |  |
| --- | --- | --- | --- | --- | --- | --- | --- | --- | --- | --- |
|  | BETA | SE | p-value | BETA | | SE | | p-value | |  |
| *sp*(A$ge$)*GRS*EBF | -0.1239 | 0.0663 | 0.0619 | -0.2117 | | 0.0815 | | 0.0094 | |  |
| *sp*(${Age}^{2}$)*GRS*EBF | 0.4914 | 0.2881 | 0.0881 | 0.7102 | | 0.2910 | | 0.0147 | |  |
| *sp*(${Age}^{3}$)*GRS*EBF | -0.7891 | 0.4887 | 0.1064 | -0.9526 | | 0.4027 | | 0.0180 | |  |
| *sp*(${Age}_{\mathcal{K}_{1}}^{3}$)*GRS*EBF | 0.8640 | 0.5599 | 0.1228 | 1.1994 | | 0.5257 | | 0.0225 | |  |
| *sp*(${Age}_{\mathcal{K}_{2}}^{3}$)*GRS*EBF | -0.0747 | 0.0792 | 0.3455 | -0.2470 | | 0.1277 | | 0.0530 | |  |
| *sp*(${Age}_{\mathcal{K}_{3}}^{3}$)*GRS*EBF | -0.0006 | 0.0008 | 0.4966 | 0.0004 | | 0.0006 | | 0.4702 | |  |
|  | | | | |  | |  | |  | |
|  | Boys | | | Girls | | | | | |  |
|  | BETA | SE | *p*-value | BETA | | SE | | *p*-value | |  |
| *sp*(A$ge$)*GRS*BF | -0.0813 | 0.0222 | 0.0003 | -0.0539 | | 0.0264 | | 0.0410 | |  |
| *sp*(${Age}^{2}$)*GRS*BF | 0.3290 | 0.0967 | 0.0007 | 0.1846 | | 0.0941 | | 0.0497 | |  |
| *sp*(${Age}^{3}$)*GRS*BF | -0.5329 | 0.1640 | 0.0012 | -0.2470 | | 0.1302 | | 0.0578 | |  |
| *sp*(${Age}_{\mathcal{K}_{1}}^{3}$)*GRS*BF | 0.5879 | 0.1879 | 0.0018 | 0.3096 | | 0.1700 | | 0.0686 | |  |
| *sp*(${Age}_{\mathcal{K}_{2}}^{3}$)*GRS*BF | -0.0550 | 0.0265 | 0.0383 | -0.0626 | | 0.0414 | | 0.1306 | |  |
| *sp*(${Age}_{\mathcal{K}_{3}}^{3}$)*GRS*EBF | -0.0001 | 0.0003 | 0.8371 | -0.0001 | | 0.0002 | | 0.6756 | |  |

Reviewer #3: In this paper, the authors examine whether exclusive or any breastfeeding up until 5 months of age is protective against later BMI gain by BMI-associated genetic risk. The paper is in general well-written and the analyses are performed satisfactorily. Additionally, the findings are of broad public interest, and the conclusions are appropriate.

We thank the reviewer for his very positive comments.

I do have some minor comments:

1. It is fine to use only 97 BMI-associated variants, but then this choice should be justified, given that over 900 are now published that explain ~6% of the variance (Yengo HMG 2018). This should especially be considered in the Discussion, and perhaps expanding the GRS could be a "future direction".

We added this paragraph in the conclusion:

“Our GRS definition was based on 94 SNPs including 69 SNPs from Locke et.al., 2015 [6]. As large meta-GWASs on BMI and obesity-related traits are fast developing, some extended definitions of GRSs are emerging such as Yengo et.al. 2018 [19] and Khera et.al, 2019 [13]. However, the correlation between BMI and GRS in the Health and Retirement Study participants derived from Locke at.al., 2015 is near identical to the correlation between the BMI and GRS derived from Yengo et.al., 2018 (r = 0.22) [32]. We are planning to generalize our study to these new GRS definitions in the near future. Also, our GRS definition is mainly based on SNPs found associated with BMI in adults and could be extended to include genetic variants more specific to children, taking advantage on recent GWAS discoveries [33-35]

33. Helgeland Ø, Vaudel M, Juliusson PB, Lingaas Holmen O, Juodakis J, Bacelis J, Jacobsson B, Lindekleiv H, Hveem K, Lie RT, Knudsen GP, Stoltenberg C, Magnus P, Sagen JV, Molven A, Johansson S, Njølstad PR. Genome-wide association study reveals dynamic role of genetic variation in infant and early childhood growth. Nat Commun. 2019 Oct 1;10(1):4448.

34. De Silva N, S S, AC A, U S, S D, Taal R, et al. Genetic architecture of early childhood growth phenotypes gives insights into their link with later obesity. bioRxiv. 2017;150516.

35.Couto Alves A, De Silva NMG, Karhunen V, Sovio U, Das S, Taal HR, et al. GWAS on longitudinal growth traits reveals different genetic factors influencing infant, child, and adult BMI. Sci Adv. 2019;5(9):eaaw3095.

2. The abstract could use some clarification. Obesity is now better understood compared to what? I also find the second sentence unclear. Please also briefly add some detail about the GRS used in this study in the abstract, for example, how many SNPs, associated with adult or childhood BMI originally, etc.

We changed to first 2 sentences into: “Recent discoveries from large-scale genome-wide association studies allow to explain a larger proportion of the genetic variability to BMI and obesity. The genetic risk associated with BMI and obesity can be assessed by an obesity-specific genetic risk score (GRS) constructed from genome-wide significant genetic variants.”

The number of SNPs associated with adult BMI and childhood BMI is now also indicated in this abstract.

3. Is there a word "and" missing in the final sentence of the abstract, "EBF influences early life growth AND development"?

"AND" has been added.

4. It seems an overstatement to say that EBF plays a "critical role" based on this study's findings (only a 1.4kg/m2 decrease in BMI in boys at 18), but certainly EBF is important and has lifelong benefits on BMI gain, especially for those with high genetic load of increasing alleles.

We removed "critical".

5. In the author summary, saying something is "perfect" is very subjective. Please rephrase using objective language.

We changed “perfect candidate” to “key candidate” in the abstract but left “perfect” in the introduction since we are quoting the WHO statement.

6. Author summary, last sentence: please change "susceptibility alleles" to "increasing alleles"

This has been changed.

7. Introduction, line 75: It is confusing and misleading to say that BF extends "beyond healthy children, e.g., in children with higher genetic risks". Kids with GRS for high BMI can still be healthy.

We removed "beyond healthy children".

8. Throughout the paper, please say "pediatric BMI" instead of "child BMI" since you are also talking about adolescents.

This has been changed.

9. Page 5, line 87: please add whether the GRS was weighted or unweighted

We added "weighted" before GRS.

10. Line 90: At what age did the GRS explain 1.5% of BMI variability?

At 6 years of age. This was added.

11. Page 8, line 146: Change "principle" to "principal"

The related sentence has been removed.

12. I question whether it makes sense to describe the equivalence of a 1-unit increase in GRS in terms of NUMBER of effect alleles carried given that the GRS was weighted.

Given the rapid development of GWASs, the number of SNPs associated with BMI and obesity has increased dramatically in the last 2 years, from under 100 to over 900 (Yengo et.al., 2018) and even 2.1 millions (khera et al. 2019) . Therefore, the effect of 1-unit increase in GRS on BMI might differ given the number of SNPs used to define the GRS. Instead, assessing an increase in GRS percentiles, deciles, or quartiles, etc., can provide a relative measure of GRS effect. An example has been given by Tyrell et.al [16] who presented both effects, i.e., GRS deciles and number of alleles. Following the same idea, we presented GRS effect in terms of quartile increase and calculated the number of alleles that matches a decile GRS effect, e.g., 1-decile increase in the GRS corresponds to a 4.6-allele effect in boys and 5.2 allele-effect in girls.

13. Page 9, line 174: "maternal preconception" is perhaps missing a word?

We added "BMI" after "preconception".

14. Line 173: categorize --> categorizeD
Done

15. I think Table 2 and 3 would be easier to grasp with graphical representation, and put the data in these tables in the supplement. Why not give exact p-values in the table? Also please check the formatting-- there should be a space after a numeral and before a parenthesis.

We replaced the tables 2 and 3 with corresponding figures and moved them to Appendix as tables S2 and S5. Only the P-values less than 10^-4^ were given as below this threshold since they cannot be computed accurately. Tables were reformatted as requested.

16. Discussion, line 284: "shed lights" --> shed light

Done

17. Sentence on line 284 is confusing. I would remove "The value of" and "in life" -- it is clearer without those

They have been removed.

18. Line 291: it --> they	Done

19. Top of page 16, please discuss why this GRS was used and not most recent data from Yengo et al included.

We have added the following sentence to the discussion:

“Our GRS definition was based on 94 SNPs including 69 SNPs from Locke et.al., 2015 [6]. As large meta-GWASs on BMI and obesity-related traits are fast developing, some extended definitions of GRSs are emerging such as Yengo et.al. 2018 [19] and Khera et.al, 2019 [13]. However, the correlation between BMI and GRS in the Health and Retirement Study participants derived from Locke at.al., 2015 is near identical to the correlation between the BMI and GRS derived from Yengo et.al., 2018 (r = 0.22) [32]. We are planning to generalize our study to these new GRS definitions in the near future. Also, our GRS definition is mainly based on SNPs found associated with BMI in adults and could be extended to include genetic variants more specific to children, taking advantage on recent GWAS discoveries [33-35].”

20. Line 301: "essential fluid" sounds odd

The term "fluid" has been removed.

21. Finally, the English is overall good but requires careful editing by a native speaker. There are many small mistakes throughout.

The MS was proofread by a native speaker (co-author Steve Lye).
